# The Effect of Ferroptosis-Related Genes on Prognosis and Tumor Mutational Burden in Hepatocellular Carcinoma

**DOI:** 10.1155/2021/7391560

**Published:** 2021-08-18

**Authors:** Hao Zhang, Renzheng Liu, Lin Sun, Weidong Guo, Xiao Hu

**Affiliations:** ^1^Department of Hepatobiliary Pancreatic Surgery, The Affiliated Hospital of Qingdao University, Qingdao, Shandong, China; ^2^Department of ICU, The Affiliated Hospital of Qingdao University, Qingdao, Shandong, China

## Abstract

In this study, we constructed the ferroptosis-related genes diagnostic and prognostic models. We analyzed the relationship between ferroptosis and tumor mutational burden in hepatocellular carcinoma (HCC). Eighty-four ferroptosis-related genes were analyzed by Cox regression and the least absolute shrinkage and selection operator method. Seven genes (SLC7A11, ACSL3, ACACA, SLC1A5, G6PD, ACSL6, and VDAC2) were used to construct models. The reliability of the model was verified by using the data from the ICGC database. Differential genes in high and low-risk groups revealed enrichment of many immune features by Gene Ontology and Kyoto Encyclopedia of Genes and Genomes. The degree of ferroptosis was negatively correlated with tumor mutational burden (i.e., the higher the degree of ferroptosis, the lower the tumor mutational burden). The tumor mutational burden was negatively correlated with survival. We also found that ALB, TP53, and DOCK2 may be a bridge between ferroptosis and tumor mutational burden. The reported models and the relationship with tumor mutational burden indicate new possibilities for individualized treatment of HCC patients.

## 1. Introduction

Hepatocellular carcinoma (HCC) is a liver tumor with a poor prognosis. It is the sixth most common malignancy worldwide [1], which occurs most often in those with chronic liver diseases such as cirrhosis caused by hepatitis C or B infection, primary biliary cholangitis or alcoholic hepatitis, metabolic disorders, and ingestion of aflatoxin-contaminated food that are all considered as risk factors contributing to the development of HCC [2–4]. Surgery and liver transplantation, followed by chemotherapy and radiotherapy, are considered as the main treatment approaches; the treatment is usually based on the tumor stage and size. Still, the long-term survival of patients remains poor [5]. Resistance to targeted therapies, often caused by the signaling pathway's reactivation, is one of the main reasons for poor prognosis. To complicate matters, the reason for the high refractoriness of HCC is poorly understood [6, 7]. Thus, there is an urgent need to analyze HCC from a different perspective.

Over recent years, alternative therapies have been investigated. For example, drugs that induce nonapoptotic cell death provide new opportunities for cancer treatment and limit the survival of drug-resistant clones [8]. Ferroptosis is a new mechanism that can induce cancer cell death, by increasing iron and oxidative stress [9]. Yet, the exact mechanism of action (e.g., genes involved in ferroptosis) remains unclear.

Tumor mutational burden (TMB), defined as the number of somatic mutations per megabase of an interrogated genomic sequence, varies across patients [10]. TMB has been suggested as a new biomarker for predicting the effect of the treatment of immune checkpoint inhibitors (ICIs) [11–13]. Although increasing studies have shown that TMB is important in immunotherapy, research on TMB and ferroptosis interaction is still relatively insufficient.

**Figure 1:**
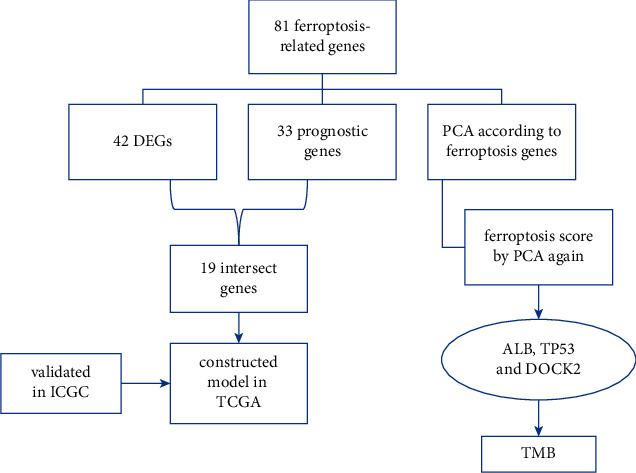
The workflow of this study.

In this study, we used data from open databases to analyze the genome of HCC and thousands of molecular targets, identify a ferroptosis-related model, and explore the relationship with TMB in HCC. Our study may be helpful for the diagnosis and treatment of HCC (Figure 1).

## 2. Materials and Methods

### 2.1. Acquisition of Ferroptosis-Related Genes

In KEGG pathway database (https://www.genome.jp/kegg/pathway.html), we found ferroptosis-related genes according to the ferroptosis pathway (map04216). Ferroptosis-related genes were found in the WP_FERROPTOSIS gene set and downloaded from GSEA database (https://www.gsea-msigdb.org/gsea/index.jsp). As no clear gene set has yet been found to be related to ferroptosis, we searched the literature related to ferroptosis, carried out a comprehensive analysis, eliminated some repetitive and unrelated genes, and added new genes with a high correlation with ferroptosis.

### 2.2. Download Transcriptome Data and Differentially Expressed Analysis

We downloaded the expression data of the hepatocellular liver carcinoma project rectified to fragments per kilobase million (FPKM) and simple nucleotide variation data of HCC in TCGA (https://tcga-data.nci.nih.gov/tcga/). Clinical data of the study were downloaded from the LIHC project in TCGA. We annotated the data by gene transfer format (GTF) files obtained from Ensembl (http://asia.ensembl.org). The expression data of the hepatocellular carcinoma were downloaded from International Cancer Genome Consortium (ICGC) (https://dcc.icgc.org/). Next, we distinguished the ferroptosis-related genes, and differentially expressed analysis was performed to screen ferroptosis-related genes in R software (|logFC| > 0.75, FDR < 0.05).

### 2.3. Establishment and Validation of a Prognostic, Predictive Signature

We screened genes related to survival; genes with a *p* value <0.05 that were considered statistically significant by univariate Cox regression analysis were incorporated into the subsequent LASSO Cox regression. Finally, we built a prognostic signature. The prognostic risk score was determined using the coefficient and the expression levels of the genes. Median was used to determine the high-risk group and low-risk group. Kaplan–Meier survival curves and time-dependent receptor operating characteristic (ROC) curves were performed to evaluate the predictive performance of the prognostic signature. The RiskScore of each sample was also visualized by R software. Then, to evaluate the clinical application value of the constructed model, we performed univariate and multivariate Cox regression analyses between the RiskScore and clinicopathological value to verify whether the model can be used as an independent clinical prognostic predictor (<0.001 = ^*∗∗∗*^, <0.01 = ^*∗∗*^, and <0.05 = ^*∗*^).

### 2.4. Gene Cluster and Ferroptosis Score

The principal component analysis was used to group genes and samples. Boruta package was used to find feature genes from ferroptosis-related genes. A group genes and B group genes were scored in each group by principal component analysis using the following formula:

(1) $$\begin{array}{c} \text{Ferroptosis score }\left(\text{Fer score}\right):\text{score of A group}-\text{score of group B}. \end{array}$$

### 2.5. Analyzing the Gene Ontology and the Kyoto Encyclopedia of Genes and Genomes

To study the function of related genes, clusterProfiler, org.Hs.eg.db, enrichplot, and ggplot2 package visualization were performed in R software.

## 3. Results

### 3.1. A Prognosis Model of Ferroptosis-Related Genes

**Figure 2:**
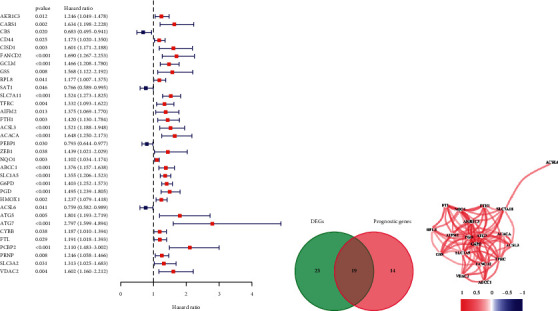
Establishing the prognosis model. (a) Selecting prognostic genes by single factor Cox analysis. (b) The Venn diagram of the intersection of DEGs and prognostic genes. (c) The correlation of 19 ferroptosis-related genes (cutoff = 0.2).

**Figure 3:**
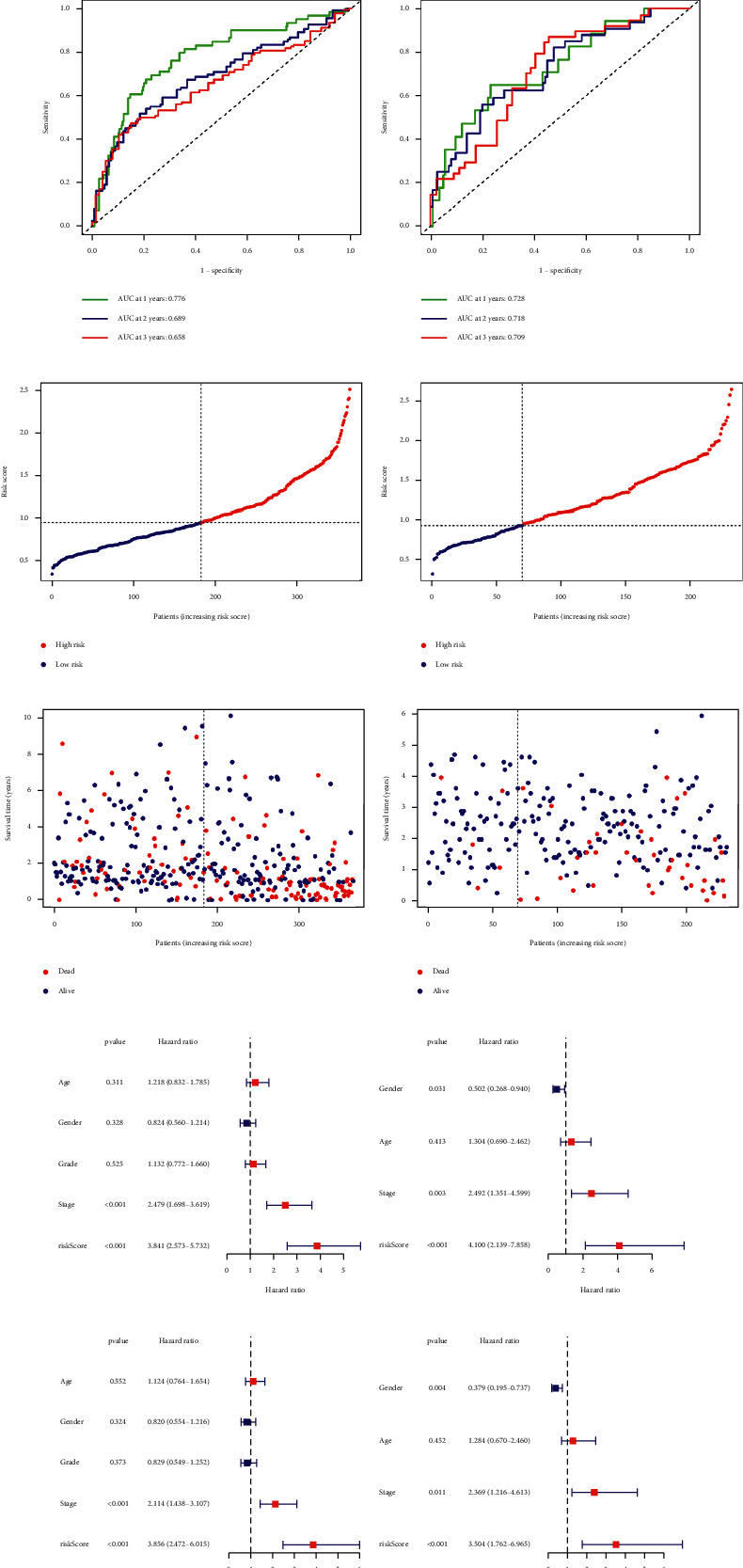
To verify the reliability of the prognosis model, patients in the high-risk group experienced a shorter survival time tested by the Kaplan Meier test (a, b), the ROC curves (c, d), the RiskScore, and SurvStat of each sample (e–h). Risk score presented as an independent prognostic predictor by univariate (i, j) and multivariate (k, l) Cox analyses in TCGA and ICGC groups. The GO (m) and KEGG (n) of the differential genes are according to high and low-risk groups of patients.

Eighty-four ferroptosis-related genes were found. Forty-two differentially expressed genes (DEGs) were screened according to the difference in their expression between cancer and normal tissues (|logFC| > 0.75, FDR < 0.05). Thirty-three genes from the 81 genes were obtained by single factor Cox at the same time (Figure 2(a)), after which we analyzed the intersection of these two groups (Figure 2(b)) and obtained 19 ferroptosis-related genes (Figure 2(c)). We analyzed these genes by the least absolute shrinkage and selection operator. We constructed a model in the TCGA database and validated the prognosis model in the ICGC database based on these seven genes (SLC7A11, ACSL3, ACACA, SLC1A5, G6PD, ACSL6, and VDAC2). The survival probability of the high-risk group was significantly lower than that of the low-risk group in the TCGA and ICGC database (Figures 3(a) and 3(b)). The AUC of ROCs curve was higher than 0.7 in the first year (Figures 3(c) and 3(d)). The RiskScore and SurvStat of each sample are shown in Figures 3(e)–3(h)).

Next, we performed a series of univariate (Figures 3(i) and 3(j)) and multivariate Cox analyses (Figures 3(k) and 3(l)) to investigate the relationship between the risk of hepatocellular carcinoma and clinicopathological characteristics. The clinical stage and risk score showed statistical differences and were presented as independent prognostic predictors. We obtained a series of differential genes according to the high and low-risk groups of patients and analyzed the Gene Ontology (GO) (Figure 3(m)) and the Kyoto Encyclopedia of Genes and Genomes (KEGG) (Figure 3(n)) for these differential genes.

### 3.2. Principal Component Analysis of the Ferroptosis-Related Genes

**Figure 4:**
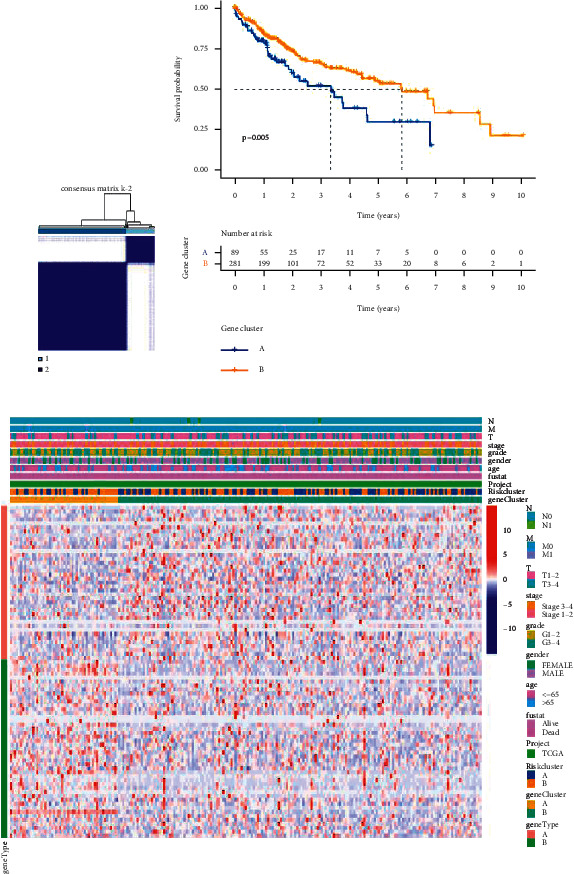
Analyzing ferroptosis-related genes by principal component analysis. (a) Consensus clustering matrix for *k* = 2. (b) Kaplan–Meier curves of survival in two clusters (cluster1/2). (c) Heatmap of clinicopathologic features of the gene cluster and risk cluster.

Next, we used ferroptosis-related related genes to type the gene expression matrix downloaded from the TCGA database and 81 ferroptosis-related genes (Figure 4(a)). We found a significant difference in survival probability between the two groups (Figure 4(b)), after which we performed a series of chi-square tests to investigate the relationship between the risk of prognosis, gene cluster, and clinicopathological characteristics (Figure 4(c)).

### 3.3. Looking for and Analyzing Feature Ferroptosis-Related Genes

**Figure 5:**
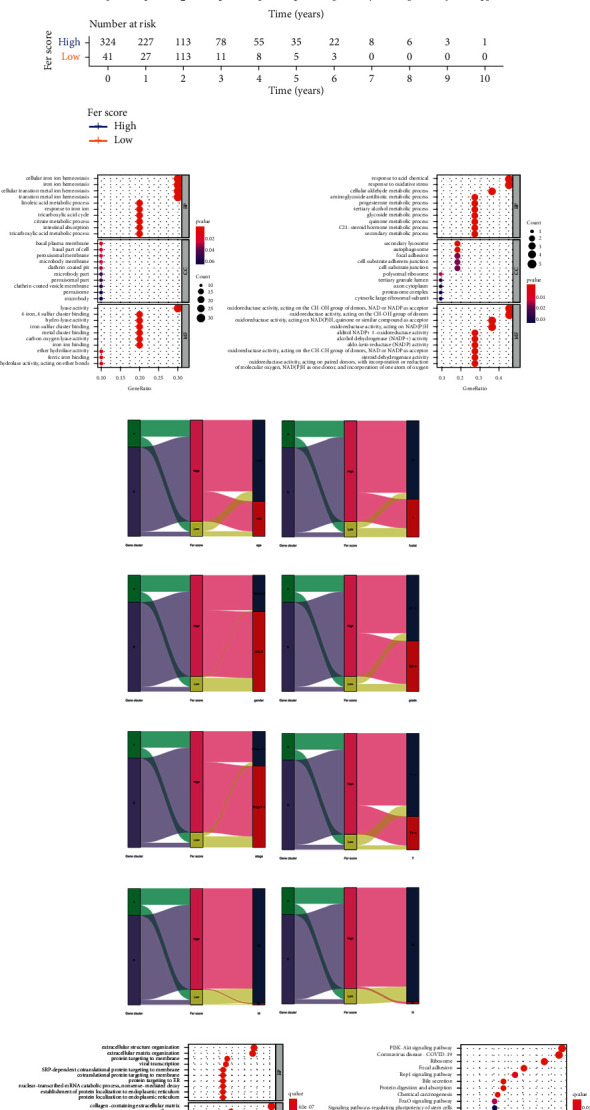
Confirming and analyzing feature ferroptosis-related genes. (a) Kaplan–Meier curves of survival in high and low Fer score groups. (b, c) GO of feature genes in A and B clusters. (d–k) The Sankey diagram of gene cluster, Fer score group, and clinical characteristics of the samples. (l, m) The GO and KEGG of DEGs in high and low-score groups.

An optimal cutoff point (−3.335958) was obtained according to the Fer score of different samples and samples were divided into the low and high-score groups. Kaplan–Meier analysis showed that patients in the low-score group had a shorter survival time than those in the high-score group (*p* < 0.001) (Figure 5(a)). Next, we analyzed the Gene Ontology (Figures 5(b) and 5(c)) in groups A and B of these feature genes. We described the gene cluster, Fer score group, and clinical characteristics of the samples by Sankey diagram (Figures 5(d)–5(k)). Finally, a series of differential genes were obtained according to the high and low-score groups of patients, and Gene Ontology (Figure 5(l)) and the Kyoto Encyclopedia of Genes and Genomes (Figure 5(m)) were analyzed for these differential genes.

### 3.4. Verification of Fer Score in Clinical Features

**Figure 6:**
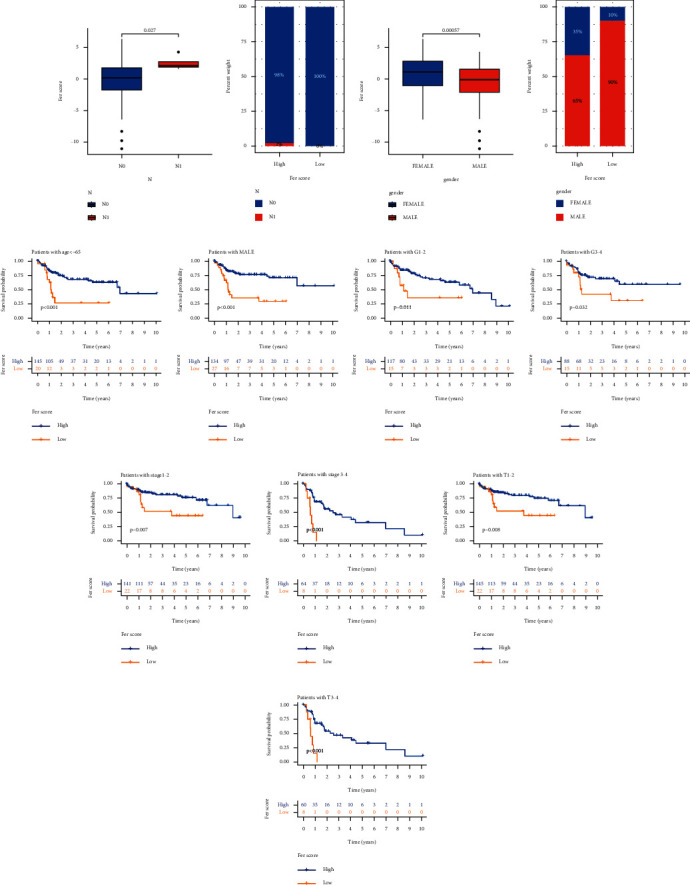
Clinicopathological features used to verify the reliability of Fer score. Discrimination of Fer score in N stage (a, b) and gender (c, d). Survival probability was different when the patients were <65 years old (e), patients were male (f), between high and low Fer score groups. The survival of different tumor grades (g, h), clinical stages (i, j), and T stage (k, l).

We studied the correlation between different groups and clinicopathological features, where N stage (Figures 6(a) and 6(b)) and gender (Figures 6(c) and 6(d)) showed statistical differences. Kaplan–Meier analysis revealed that survival probability was different in patients younger than 65 years old (Figure 6(e)) and male patients (Figure 6(f)), as well as between high and low Fer score groups. The survival of different tumor grades (Figures 6(g) and 6(h)), clinical stages (Figures 6(i) and 6(j)), and T stage (Figures 6(k) and 6(l)) was significantly related to different groups.

### 3.5. The Effect of Ferroptosis on Tumor Mutational Burden

**Figure 7:**
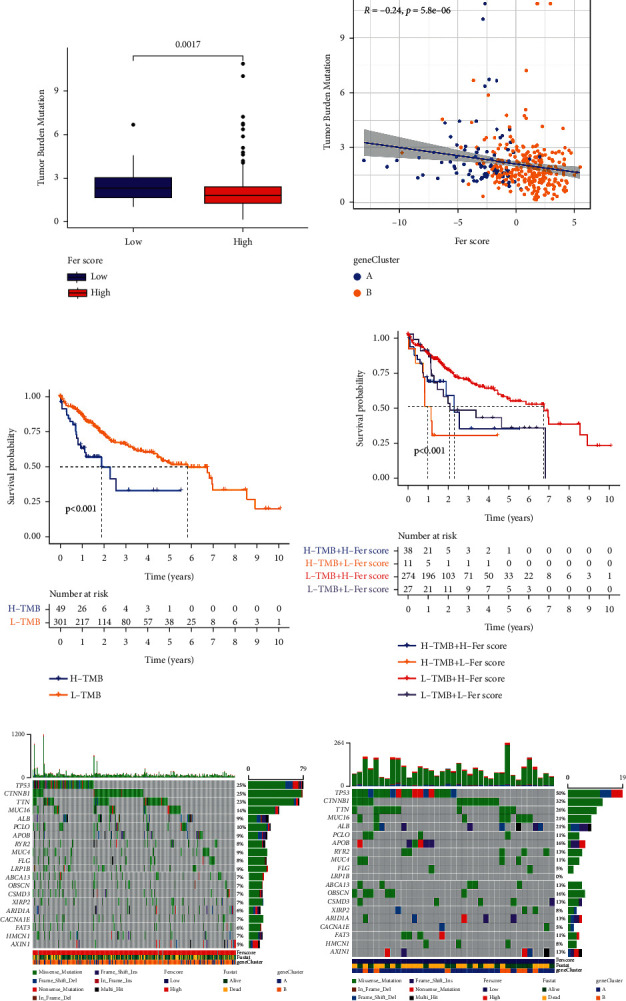
The effect of Fer score on TMB. (a-b) The relationship of TMB and Fer score. The effect of TMB on survival (c), Fer score, and TMB on survival (d). The oncoplots of high (e) and low (f) Fer score groups.

We found that the high Fer score group was associated with a lower tumor mutational burden than the low Fer score group (Figure 7(a)). Next, we described the association between Fer score and tumor mutational burden based on gene cluster (Figure 7(b)). There was a negative correlation between Fer score and tumor mutational burden (*R* = −0.24, *p* < 0.001). We got the optimal cutoff point according to the TMB point of samples and grouped the samples according to the cutoff point. Our results revealed that the survival probability of the high tumor mutational burden group was significantly lower than that of the low tumor mutational burden group (Figure 7(c)). We also conducted a combined survival analysis of Fer score and tumor mutational burden. We discovered that the survival probability of the high tumor mutational burden and low Fer score group was the lowest, while the survival probability of the low tumor mutational burden and high Fer score group was the highest (Figure 7(d)). We described the different gene mutations form in the high (Figure 7(e)) and low (Figure 7(f)) Fer score groups and studied the differences of wild type and mutation of different genes in the high and low Fer score groups by using the chi-square test. Our results revealed that the mutation of ALB (*p*=0.047), TP53 (*p*=0.002), and DOCK2 (*p*=0.012) was significantly different between high and low Fer score groups.

## 4. Discussion

Several drugs have been found to induce ferroptosis, such as erastin [14], sulfasalazine [15], artemisinin [16], sorafenib [17], and capecitabine [18–21]. A number of genes associated with ferroptosis have also been reported. In this study, we found 81 ferroptosis-related genes from databases. The ferroptosis-related genes with a different expression and the survival-related genes obtained by single factor Cox regression analysis were taken as the intersection in the TCGA group. Next, we analyzed these genes by LASSO analysis and calculated the risk score for each patient according to the median risk score of the TCGA group, TCGA group, and ICGC group. Finally, patients were divided into high-risk group and low-risk group (risk score = SLC7A11*∗* 0.1674 + ACSL3 *∗* 0.0001 + ACACA*∗*0.1593 + SLC1A5 *∗* 0.0830 + G6PD *∗* 0.1318 + ACSL6 *∗* −0.0596 + VDAC2*∗*0.0105). The survival curves, ROC curves, RiskScore, and SurvStat plot of the high-risk and low-risk groups in TCGA and ICGC databases were analyzed. Univariate and multivariate Cox regression analyses revealed that this model could be used as an independent prognostic predictor to investigate the relationship between the risk of hepatocellular carcinoma and clinicopathological characteristics. Different genes were found between high and low-risk groups (|logFC| > 1, FDR < 0.05), and the Gene Ontology and the Kyoto Encyclopedia of Genes and Genomes were analyzed for these differential genes.

Next, we grouped 81 ferroptosis-related genes by principal component analysis. In addition, the survival differences between the two groups were analyzed. We used the Boruta package to find feature genes from ferroptosis-related genes. Each group was scored again by principal component analysis. Next, we found a link between Fer score and TMB. Our results also showed that ALB, TP53, and DOCK2 may be a bridge between ferroptosis and tumor mutational burden. ALB encodes albumin protein, which regulates the colloidal osmotic pressure of blood [22]. ALB is widely known as a prognostic indicator of gastric cancer. TP53 is a well-known tumor suppressor gene involved in regulating the cell cycle and negative regulation of cell division by a series of genes [23, 24]. Recent studies have found that TP53 can have a role in the process of ferroptosis [25, 26]. Numerous studies have taken TP53 as a marker gene of ferroptosis. DOCK2 encodes the dedicator of cytokinesis 2 protein, which is mainly expressed in peripheral blood immune-related cells and is involved in changes of cytoskeleton proteins when chemokines act on lymphocytes [27, 28].

In summary, we constructed a model consisting of seven ferroptosis-related genes, which have a great predictive value in HCC. These models and the relationship with tumor mutational burden indicate new possibilities for individualized treatment of HCC patients.

This study has a few limitations. The dataset used for the analysis was not relatively sufficient as it was downloaded from TCGA and ICGC. In addition, due to the lack of corresponding tissue samples and follow-up clinical data, our theory is difficult to be verified in the current situation. However, verification from different perspectives was used to confirm the findings of this study. Based on these analyses, we assume that our model is meaningful despite the lack of clinical validation data. Therefore, we will continue to seek clinical samples and expand the sample scale for future tests. We hope that our study can have a pivotal role in the treatment of liver cancer.

## 5. Conclusion

This study has a few limitations. The dataset used for the analysis was not relatively sufficient as it was downloaded from TCGA and ICGC. In addition, due to the lack of corresponding tissue samples and follow-up clinical data, our theory is difficult to be verified in the current situation. However, verification from different perspectives was used to confirm the findings of this study. Based on these analyses, we assume that our model is meaningful despite the lack of clinical validation data. Therefore, we will continue to seek clinical samples and expand the sample scale for future tests. We hope that our study can have a pivotal role in the treatment of liver cancer.

## Data Availability

The datasets used and/or analyzed during the current study are available from the corresponding author upon request.

## Abbreviations

HCC: Hepatocellular carcinoma
TMB: Tumor mutational burden
ICIs: Immune checkpoint inhibitors
FPKM: Fragments per kilobase million
GTF: Gene transfer format
ICGC: International Cancer Genome Consortium
ROC: Receptor operating characteristic
Fer score: Ferroptosis score
DEGs: Differentially expressed genes
GO: Gene Ontology
KEGG: Kyoto Encyclopedia of Genes and Genomes.
